# YASA automated sleep staging performance across seven nights of normal sleep and sleep restriction

**DOI:** 10.1007/s44470-026-00059-x

**Published:** 2026-04-08

**Authors:** Zhiyi Chen, Natalya Pruett, Michael K. Scullin

**Affiliations:** Department of Psychology and Neuroscience, Baylor University, One Bear Place 97334, Waco, TX 76798 USA

**Keywords:** Automatic sleep staging, YASA, Sleep restriction, Sleep scoring, Machine learning, PSG

## Abstract

**Study objectives:**

This study aimed to compare YASA’s automated sleep staging to manual staging in the context of a multi-night experimental sleep restriction protocol.

**Methods:**

Seventy-five adults (58% female; 57% nonwhite) participated in up to seven nights of laboratory-based polysomnography measurements. The study involved one adaptation night, followed by three nights of normal sleep (9 h time in bed) and three nights of sleep restriction (5.5 h time in bed). Condition order was counterbalanced. Manual sleep staging was performed by a registered polysomnographic technician. Sleep data were exported and processed using Python 3.12, MNE 1.8.0, and YASA 0.6.5 for automated sleep staging.

**Results:**

Across 483 valid sleep nights, there was 82.9% overall agreement between YASA scoring and manual scoring. Stage-specific agreement was 40.3% (N1), 85.1% (N2), 86.9% (N3), and 78.7% (REM). Agreement was higher during normal sleep nights than sleep restriction nights, particularly for wake, N1, and REM sleep classifications.

**Conclusions:**

YASA-based staging exhibited good overall agreement with manual scoring during normal sleep nights. However, caution is needed when interpreting N1 estimates, as well as in interpreting data during short sleep nights.

**Brief summary:**

**Current knowledge/study rationale:**

Sleep stage scoring is typically conducted manually by at least one experienced technician, but this process is laborious and subject to biases. This study investigated whether a machine-learning-based open tool—YASA—could automatically stage polysomnography data with acceptable accuracy.

**Study impact:**

Across 430,813 epochs, YASA showed acceptable accuracy in sleep staging, making it an efficient tool for the sleep community. However, caution is still needed for some applications, such as interpreting YASA’s N1 estimates as well as data from short sleep nights.

## Introduction

### Traditional manual sleep staging

Sleep staging is fundamental to sleep research and clinical sleep medicine. Historically, researchers began to quantify human sleep physiology in the early to mid-twentieth century [1–3], but standardization in the analytical approach to polysomnography recordings only emerged with the Rechtschaffen and Kales manual in 1968 [4]. Though elements of the Rechtschaffen and Kales manual persist in contemporary approaches, most clinics and laboratories currently follow American Academy of Sleep Medicine guidelines (AASM) [5, 6]. Based on AASM guidelines, electroencephalography (EEG), electrooculography (EOG), and electromyography (EMG) are used to visually identify five stages (Wake, N1, N2, N3, and REM) for each 20–30 s epoch of data. This method is generally considered to be the gold standard for sleep staging.

Manual sleep staging is widely adopted, but has well-known limitations. Specifically, this approach requires considerable training for sleep technicians to perform visual inspection of each epoch, a process that introduces risks for subjective biases and requires 2–3 h of labor for a single night’s data [7]. Even for experienced sleep technicians, variability between raters is unavoidable, which limits standardization across laboratories. The manual scoring inter-rater agreement for experienced technicians is 83%, with the lowest consistency occurring for N1 (63%) and the highest for REM sleep (91%) [8].

### Potential advantages of automated sleep staging and validation gaps

Automated sleep staging tools have recently emerged as a potential alternative to manual sleep staging [9–11]. If effective, these tools would provide standardized outputs, eliminate subjective biases that are inherent in manual scoring, and reduce both labor and time costs. Machine learning-based algorithms such as Yet Another Spindle Algorithm (YASA) [12, 13] classify epochs into sleep stages by extracting EOG amplitude variations, EMG activity features, EEG time-domain features (e.g., standard deviation and skewness), and EEG frequency-domain features (e.g., spectral power and power ratios). The agreement rates between automated staging algorithms and manual sleep staging have been moderate to high in retrospective dataset analyses (YASA [12, 13]; Z3Score [14]; Stanford-stage algorithm [15, 16]; SeqSleepNet algorithm [17]), sometimes approaching the level of inter-rater consistency typically observed amongst expert human technicians (i.e., 82–83%) [8, 18]. These are promising outcomes but require further testing in independent datasets and under varying conditions.

### Research objectives

The goal of this study was to provide an independent evaluation of YASA’s performance across seven nights of adaptation, normal sleep, and sleep restriction conditions. Sleep restriction is a useful context for investigating YASA’s performance because this manipulation is commonly used in sleep research, it is known to change sleep macroarchitecture (increased N1, reduced wake, N2, and REM sleep) [19], and there have been concerns that short sleep can challenge some sleep algorithms [20–22]. On an epoch-by-epoch level, we compared YASA performance to expert manual scoring, setting ≥ 82% correspondence as the benchmark for acceptable overall agreement [8, 18]. In addition, we examined rates of inter-individual variability in the level of YASA-manual correspondence, YASA performance on individual sleep stages, and performance on other PSG metrics (e.g., sleep efficiency) across adaptation nights, normal sleep nights, and sleep restriction nights.

## Methods

### Participants

Participants were recruited between February 2020 and November 2023 via online SONA systems, on-campus flyers, in-person recruitment, and other advertisements. Inclusion criteria were being aged 18–29 with no diagnosed history of sleep disorders, neurological conditions, or psychiatric disorders. Participants were also required to be free of medications known to affect sleep and to report a non-extreme chronotype (i.e., scoring between 30 and 70 on the Morningness-Eveningness Questionnaire) [23]. The Baylor University Institutional Review Board approved the study, and all participants provided written informed consent. Participants received monetary compensation for completing each study session.

### Study design

Eligible participants took part in a laboratory-based sleep study involving up to seven nights of polysomnography recordings under three conditions (adaptation/baseline, normal sleep, and sleep restriction). The first night served as an adaptation night. Approximately one week later, participants completed three consecutive nights of normal sleep (10:00 PM to 7:00 AM). Following a washout period (typically four nights), participants completed three consecutive nights of sleep restriction (1:30 AM to 7:00 AM). The order of the normal sleep and sleep restriction phases was counterbalanced. Data collection also involved questionnaires and cognitive tasks, but those instruments were not included in the current report except for the Pittsburgh Sleep Quality Index (PSQI) [24], which was used to inform baseline sleep quality.

### Polysomnography

The Comet XL Plus system (Grass Technologies, West Warwick, RI) was used to record overnight sleep data. EEG signals were acquired from 18 locations (Fp1, Fp2, F3, F4, F7, F8, Fz, C3, C4, P3, P4, Pz, T3, T4, T5, T6, O1, and O2) at a sampling rate of 400 Hz, with grounding at Fpz and Cz and reference electrodes placed on the contralateral mastoids (A1, A2). In addition, recording included left (LOC) and right (ROC) EOG and mentalis EMG (“Chin1” and “Chin2”). On the adaptation night, respiratory measures were also included (nasal pressure, chest/abdominal movements, and fingertip pulse oximetry).

### Sleep staging

Manual sleep staging was performed within TWin software (Grass Technologies, West Warwick, RI) by a registered polysomnographic technician who had more than 10 years of experience. The technician identified the sleep stages for each 30-s epoch according to AASM guidelines [6].

Automated sleep staging was performed in Python version 3.12. Sleep data were exported from the TWin system as European Data format (EDF), a format that supports digital PSG data storage and exchange [25]. The raw EDF data were read by MNE version 1.8.0—a Python package used for EEG analysis and visualization. YASA version 0.6.5 was used for automated sleep staging. We selected C4-A1 as the EEG channel, LOC-A2 as the EOG channel, and Chin1-Chin2 as the EMG channel for sleep staging parameters. These parameters were based on published YASA recommendations [13].

### Statistical analysis

All data were analyzed using R version 4.4.2. First, we assessed epoch-by-epoch sleep stage agreement between human scoring (i.e., treated as the ground truth reference) and YASA automated sleep staging across all valid nights of data. In addition, the agreement rates for each sleep stage (Wake, N1, N2, N3, and REM) were calculated and expressed as percentages. To characterize individual variability, we examined the distribution and range of agreement rates between YASA and human scoring for each participant across all sleep nights. All agreement rates were derived from the sleep window between lights out and lights on, and higher values represented higher agreement rates.

Second, for the participants who completed all three conditions with valid data, we conducted inferential statistics to compare the agreement rates across these conditions. When the agreement rate data were not normally distributed (as assessed by the Shapiro–Wilk test), we used the non-parametric Friedman test to compare the adaptation/baseline, normal sleep, and sleep restriction conditions. For this analysis, agreement scores from the multiple normal sleep and sleep restriction nights were first averaged for each participant to yield a single value per condition. Significant results from the Friedman test were followed by post-hoc Wilcoxon signed-rank tests to identify specific between-condition differences. Statistical significance was determined when *p* < 0.05.

Finally, common sleep measures derived from manual scoring and YASA sleep staging were compared with non-parametric Wilcoxon signed-rank tests (due to non-normal distributions as assessed by the Shapiro–Wilk test) and Pearson correlations. Sleep measures included stage durations (Wake, N1, N2, N3, and REM in minutes), sleep onset latency, REM latency, wake after sleep onset (WASO), total sleep time (TST), sleep efficiency, and number of arousals.

## Results

Seventy-five healthy adults participated in this study (mean age = 19.95 ± 1.13 years; 55% female; 57% nonwhite; PSQI global = 4.59 ± 1.91). Sixty-seven participants completed all procedures (7 of 7 possible nights), six participants withdrew following the adaptation night (1 of 7 possible nights), one participant withdrew following an adaptation night plus one sleep restriction night (2 of 7 possible nights), and one participant was missing data from the second night of the normal sleep phase (6 of 7 possible nights). Reasons for study withdrawal included discomfort from the equipment (e.g., polysomnography), scheduling conflicts, and personal reasons. In total, there were 483 nights of valid sleep data that consisted of 75 adaptation nights, 203 normal sleep nights, and 205 sleep restriction nights.

### General sleep stage agreement

**Fig. 1:**
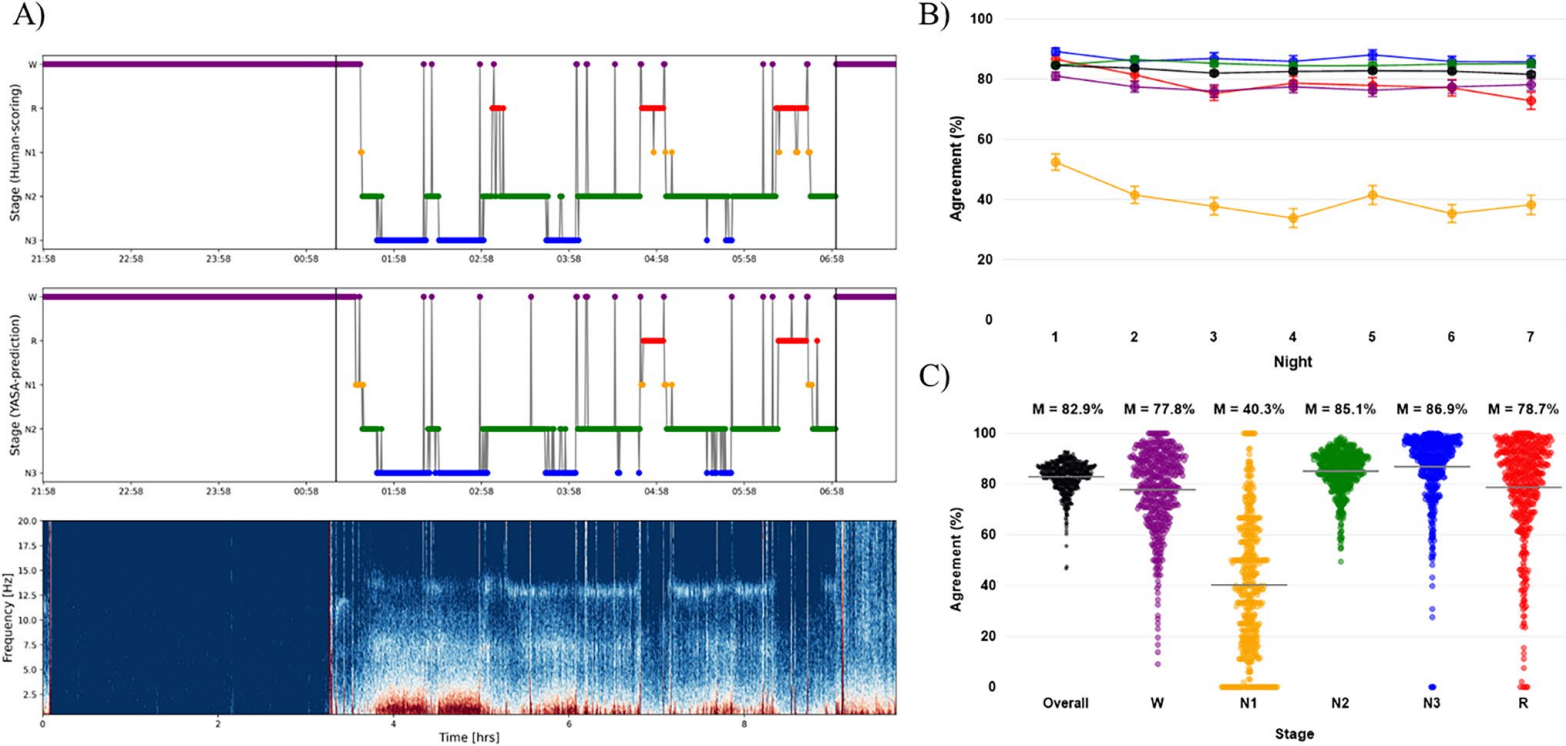
Sleep staging agreement results. **A** Representative hypnograms from one participant during a sleep restriction night. Top: Human manual staging (ground truth); Middle: YASA automated sleep staging. Vertical black lines demarcate lights-out to lights-on periods; Bottom: Corresponding spectral power plot (0.5–20 Hz). **B** Mean agreement rate across seven nights for overall (black) and five stages: Wake (purple), N1 (yellow), N2 (green), N3 (blue), and REM (red). Error bars indicate the standard error of the mean (SEM). **C** Scatter plot distribution of overall and stage-specific (Wake, N1, N2, N3, and REM) agreement rates across all 483 nights. Each dot represents a single night. The percentage number and the middle grey horizontal line indicate the mean value agreement for each category

Averaged across all nights (430,813 epochs), there was 82.90% overall agreement between YASA scoring and human manual scoring. Table 1 displays the detailed summary statistics, and Fig. 1a provides a representative hypnogram. In considering the order of nights (night 1 to night 7), Fig. 1b and Table 2 show that agreement rates were similar across nights but highest during the first night (i.e., adaptation/baseline; 84.61%). Regarding agreement rates for sleep stages, high agreement was observed for N3 (86.91%) and N2 (85.13%), moderate agreement was observed for REM (78.72%) and wake (77.82%), and low agreement was observed for N1 (40.31%). Importantly, Fig. 1c illustrates that there was inter-individual variability in YASA-manual agreement especially for N1 and REM, which ranged from individual nights with 0% agreement to nights with 100% agreement. Intraclass correlation coefficients indicated that overall agreement was not a stable trait (ICC = 0.242), but more likely to be influenced by night-specific factors. Supporting this conclusion, the inter-individual variability was not related to age, gender, race/ethnicity, or PSQI global scores (*p*s > 0.05).

**Table 1:** Sleep staging agreement across adaptation, normal sleep, and sleep restriction conditions

	All nights (*N* = 483)		Adaptation nights (*N* = 75)		Normal sleep nights (*N* = 203)		Sleep restriction nights (*N* = 205)	
Stages	Mean (SD)	Median (IQR)	Mean (SD)	Median (IQR)	Mean (SD)	Median (IQR)	Mean (SD)	Median (IQR)
Overall	82.90 (6.08)	84.11 (6.75)	84.61 (4.87)	85.97 (4.56)	83.66 (5.93)	84.87 (5.99)	81.52 (6.35)	82.58 (7.42)
W	77.82 (15.87)	80.43 (20.74)	81.11 (11.83)	83.33 (16.47)	77.73 (15.23)	80.43 (18.61)	76.69 (17.58)	80.00 (23.81)
N1	40.31 (25.44)	39.47 (40.57)	52.47 (23.55)	50.00 (35.83)	45.42 (22.16)	45.31 (30.37)	30.81 (25.90)	24.00 (38.46)
N2	85.13 (8.22)	86.71 (9.46)	84.71 (6.40)	85.19 (7.62)	86.20 (7.15)	87.31 (8.50)	84.24 (9.61)	86.59 (12.28)
N3	86.91 (14.21)	91.16 (13.30)	89.24 (10.46)	91.95 (12.11)	85.90 (15.28)	90.91 (13.44)	87.05 (14.24)	90.95 (13.63)
R	78.72 (19.97)	84.21 (20.78)	86.67 (13.88)	90.07 (15.39)	82.69 (14.21)	85.16 (16.16)	71.88 (24.22)	78.81 (28.84)

All agreement values are expressed as percentages. Data are shown as mean (standard deviation) and median (interquartile range)

**Table 2:** Sleep staging agreement across seven nights

	Night 1 (*N* = 75)	Night 2 (*N* = 68)	Night 3 (*N* = 68)	Night 4 (*N* = 68)	Night 5 (*N* = 68)	Night 6 (*N* = 68)	Night 7 (*N* = 68)
Stages	Mean (SD)	Mean (SD)	Mean (SD)	Mean (SD)	Mean (SD)	Mean (SD)	Mean (SD)
Overall	84.61 (4.87)	83.73 (5.97)	82.04 (6.83)	82.60 (5.67)	82.83 (6.03)	82.68 (5.40)	81.63 (7.31)
W	81.11 (11.83)	77.52 (14.57)	76.09 (17.23)	77.52 (16.20)	76.35 (16.76)	77.54 (18.29)	78.23 (15.87)
N1	52.47 (23.55)	41.58 (23.50)	37.81 (23.89)	33.88 (26.01)	41.55 (26.05)	35.38 (24.59)	38.27 (26.74)
N2	84.71 (6.40)	86.56 (7.33)	85.33 (9.26)	84.54 (9.71)	84.56 (8.72)	85.08 (8.24)	85.22 (7.76)
N3	89.24 (10.46)	86.03 (13.29)	86.91 (16.01)	86.00 (15.66)	88.14 (13.84)	85.95 (13.87)	85.84 (16.05)
R	86.67 (13.88)	81.50 (17.18)	75.28 (18.87)	78.74 (18.64)	77.97 (21.75)	77.17 (22.56)	72.87 (23.39)

All agreement values are expressed as percentages. Data are shown as mean (standard deviation)

### Sleep stage misclassifications

To explore the sources of sleep stage disagreements, we analyzed the confusion matrix detailing the epoch-by-epoch correspondence between manual and YASA staging (see Table 3). The primary source of error was the misclassification of stage N1. Across all nights, manually scored N1 epochs were commonly classified by YASA as either stage N2 (25.87%) or REM sleep (19.25%). YASA also sometimes classified manually scored REM as stage N2 (normal sleep: 11.27%; sleep restriction: 21.88%).

**Table 3:** Confusion matrix of YASA’s staging for each manually scored stage

		Yasa scoring					
Manual scoring		W	N1	N2	N3	R	Epochs
All nights (*N* = 483)	W	**30,974 (80.83%)**	3433 (8.96%)	2544 (6.64%)	304 (0.79%)	1067 (2.78%)	38,322
	N1	1383 (12.90%)	**4442 (41.43%)**	2774 (25.87%)	59 (0.55%)	2064 (19.25%)	10,722
	N2	3486 (1.77%)	5314 (2.70%)	**167,684 (85.17%)**	10,516 (5.34%)	9892 (5.02%)	196,892
	N3	1109 (1.09%)	46 (0.05%)	12,139 (11.98%)	**87,807 (86.68%)**	202 (0.20%)	101,303
	R	1162 (1.39%)	2879 (3.45%)	11,933 (14.28%)	189 (0.23%)	**67,390 (80.66%)**	83,553
	Total						**430,792**
Adaptation baseline (*N* = 75)	W	**9699 (83.96%)**	1081 (9.36%)	517 (4.48%)	42 (0.36%)	213 (1.84%)	11,552
	N1	420 (15.06%)	**1386 (49.71%)**	546 (19.58%)	52 (1.87%)	384 (13.77%)	2788
	N2	509 (1.39%)	1456 (3.98%)	**30,978 (84.69%)**	1744 (4.77%)	1892 (5.17%)	36,579
	N3	116 (0.77%)	13 (0.09%)	1575 (10.40%)	**13,446 (88.75%)**	0 (0.00%)	15,150
	R	133 (1.02%)	429 (3.28%)	1039 (7.95%)	23 (0.18%)	**11,449 (87.58%)**	13,073
	Total						**79,142**
Normal sleep (*N* = 203)	W	**15,512 (80.52%)**	1656 (8.60%)	1430 (7.42%)	164 (0.85%)	502 (2.61%)	19,264
	N1	622 (11.35%)	**2414 (44.06%)**	1326 (24.20%)	6 (0.11%)	1111 (20.28%)	5479
	N2	1925 (1.83%)	3045 (2.89%)	**90,485 (85.88%)**	4732 (4.49%)	5175 (4.91%)	105,362
	N3	414 (0.94%)	23 (0.05%)	5899 (13.46%)	**37,398 (85.35%)**	83 (0.19%)	43,817
	R	676 (1.58%)	1720 (4.03%)	4812 (11.27%)	68 (0.16%)	**35,409 (82.95%)**	42,685
	Total						**216,607**
Sleep restriction (*N* = 205)	W	**5763 (76.78%)**	696 (9.27%)	597 (7.95%)	98 (1.31%)	352 (4.69%)	7506
	N1	341 (13.89%)	642 (26.15%)	**902 (36.74%)**	1 (0.04%)	569 (23.18%)	2455
	N2	1052 (1.91%)	813 (1.48%)	**46,221 (84.11%)**	4040 (7.35%)	2825 (5.14%)	54,951
	N3	579 (1.37%)	10 (0.02%)	4665 (11.02%)	**36,963 (87.31%)**	119 (0.28%)	42,336
	R	353 (1.27%)	730 (2.63%)	6082 (21.88%)	98 (0.35%)	**20,532 (73.87%)**	27,795
	Total						**135,043**

Values represent the number of epochs and the corresponding row-wise percentage. Bolded values represent the majority agreement between manual and YASA staging

### Comparison of agreement rate across experimental conditions

**Fig. 2:**
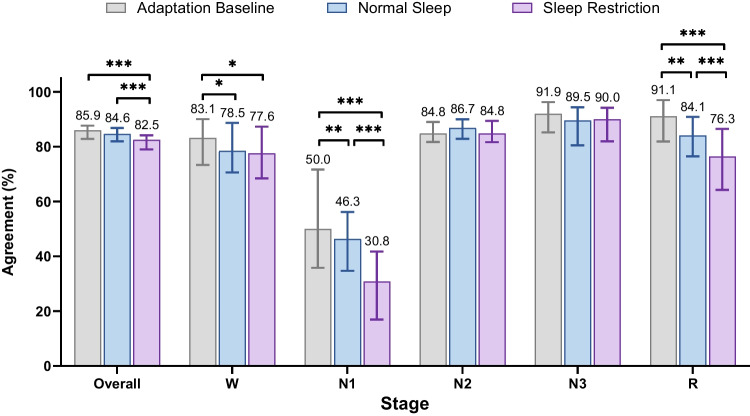
Comparative histograms between adaptation/baseline (grey), normal sleep (blue), and sleep restriction (purple) condition nights. The number represents median value, and the error bars represent the 75^th^ and 25^th^ percentile interquartile range

To compare the agreement rate between human and YASA staging across three experimental conditions, we performed Friedman tests on the agreement rates for overall performance and for each sleep stage. Figure 2 displays the distribution of overall agreement rates. This analysis was limited to the *n* = 68 participants who completed all conditions (475 nights of data).

For overall agreement, there was a significant difference across the three conditions (*χ*^2^(2) = 29.1, *p* < 0.001). Post hoc Wilcoxon signed-rank tests showed that agreement during sleep restriction nights (Mdn = 82.53%) was significantly lower than during adaptation nights (Mdn = 85.94%; *p* < 0.001, *r* = 0.54) and normal sleep nights (Mdn = 84.58%; *p* < 0.001, *r* = 0.53). No significant difference was found between adaptation and normal sleep nights (*p* = 0.281, *r* = 0.13).

A similar pattern emerged for stages with lower agreement rates (N1, REM, and Wake). For N1, agreement was highest during adaptation (Mdn = 50.00%), somewhat reduced during normal sleep (Mdn = 46.34%), and lowest during sleep restriction (Mdn = 30.84%, *χ*^2^(2) = 36.8, *p* < 0.001) (comparisons of sleep restriction versus adaptation: *p* < 0.001, *r* = 0.65; sleep restriction versus normal sleep: *p* < 0.001, *r* = 0.64). For REM, a similar decline in agreement was seen on sleep restriction nights (Mdn = 76.34%) compared to adaptation nights (Mdn = 91.14%; *p* < 0.001, *r* = 0.66) and normal sleep nights (Mdn = 84.05%; *p* < 0.001, *r* = 0.66), *χ*^2^(2) = 43.1, *p* < 0.001. For Wake, there was also a significant difference across conditions (*χ*^2^(2) = 7.15, *p* = 0.028), driven by moderately higher agreement during adaptation nights (Mdn = 83.12%) than sleep restriction nights (Mdn = 77.56%; *p* = 0.011, *r* = 0.31). Furthermore, for N1, REM, and Wake, agreement during adaptation nights was moderately higher than during normal sleep nights (N1: *p* = 0.007, *r* = 0.33; REM: *p* = 0.005, *r* = 0.34; Wake: *p* = 0.03, *r* = 0.26). There were no significant differences across the three conditions for stage N2 (*χ*^2^(2) = 5.32, *p* = 0.070) or stage N3 (*χ*^2^(2) = 3.65, *p* = 0.161).

### Sleep measurement comparison

**Fig. 3:**
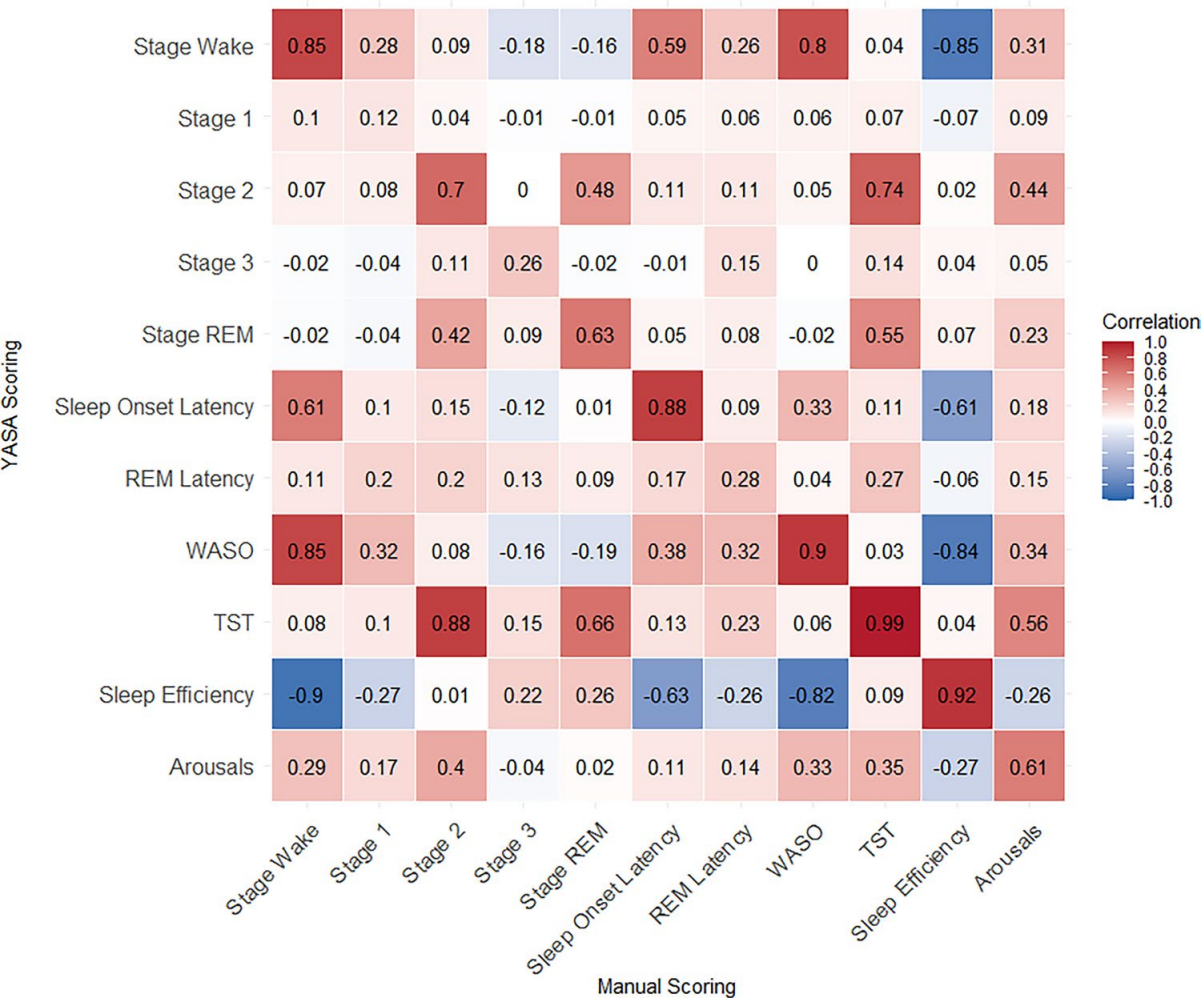
Correlation heatmap of sleep measures derived from manual and YASA scoring. The number displays the Pearson correlation coefficient, with the color indicating the direction (red for positive, blue for negative) and color intensity representing the magnitude of the correlation

Figure 3 shows the correlation heatmap for sleep measures derived from manual and YASA scoring. Some measures showed very strong correlations between systems, including TST (*r* = 0.99), WASO (*r* = 0.90), and sleep efficiency (*r* = 0.92), whereas other metrics showed low correlations (*r*s < 0.30 for stage N1 and REM latency).

Summary values from Wilcoxon signed-rank tests are shown in Table 4. Relative to manual scoring, YASA showed longer stage N1 duration (Mdn = 17.00 vs. 8.50 min; *r* = 0.54, *Z* = − 11.80, *p* < 0.001), greater WASO (Mdn = 17.50 vs. 14.00 min; *r* = 0.30, *Z* = − 6.60, *p* < 0.001), and more arousal events (Mdn = 22.00 vs. 19.00; *r* = 0.33, *Z* = − 7.20, *p* < 0.001). Conversely, YASA scoring showed shorter stage N2 duration (Mdn = 186.50 vs. 218.50 min; *r* = 0.23, *Z* = − 5.14, *p* < 0.001) and shorter sleep onset latency (Mdn = 8.00 vs. 10.05 min; *r* = 0.59, *Z* = − 12.50, *p* < 0.001). No statistically significant differences were observed in other measured parameters across the combined nights (*p*s > 0.05).

**Table 4:** Statistical results on sleep metrics across all nights (*N* = 483)

	Manual scoring		YASA scoring		Test statistics		
Measures	Mean (SD)	Median (IQR)	Mean (SD)	Median (IQR)	*r*	*Z*	*p*
Stage W duration (mins)	40.23 (40.52)	28.5 (34.25)	40.79 (41.27)	29.5 (32.5)	0.03	− 0.55	0.581
Stage N1 duration (mins)	12.98 (20.15)	8.5 (12)	30.24 (45.16)	17 (23.75)	0.54	− 11.80	< 0.001***
Stage N2 duration (mins)	201.38 (73.12)	218.5 (125.75)	180.57 (84.79)	186.5 (131)	0.23	− 5.14	< 0.001***
Stage N3 duration (mins)	104.9 (23.92)	105 (30.5)	109.75 (44.67)	106 (45.25)	0.04	− 0.99	0.323
Stage R duration (mins)	86.48 (29.75)	85.5 (42.5)	84.61 (35.12)	88.5 (47.25)	0.08	− 1.66	0.097
Sleep latency (mins)	16.01 (17.18)	10.5 (14.5)	12.57 (15.17)	8 (10.5)	0.59	− 12.50	< 0.001***
REM latency (mins)	82.41 (39.02)	72 (29.25)	77.83 (52.76)	72.5 (47.5)	0.08	− 1.66	0.097
WASO (mins)	23.66 (30.73)	14 (19.5)	26.89 (31.51)	17.5 (22)	0.30	− 6.60	< 0.001***
TST (mins)	405.78 (89.82)	435 (176.75)	406 (90.97)	436 (179.25)	0.03	− 0.75	0.453
Sleep efficiency (%)	91.63 (7.41)	93.64 (5.76)	91.6 (7.3)	93.76 (6.24)	0.02	− 0.35	0.728
Number of arousals (count)	20.68 (11.01)	19 (15)	23.77 (10.97)	22 (14)	0.33	− 7.20	< 0.001***

*p < 0.05; **p < 0.005; ***p < 0.001

## Discussion

### Overall YASA performance

Overall, YASA demonstrated satisfactory performance in automated sleep staging. Across 483 valid sleep nights comprising 430,813 epochs, YASA achieved an 82.90% agreement rate with expert manual staging. This performance level closely aligns with the 82.60% inter-rater reliability reported in large-scale studies of human scorers [8], where approximately 2500 raters scored 1800 epochs. There was, however, considerable heterogeneity in outcomes across individuals, sleep stages, and normal versus sleep-restricted conditions. In this section, we consider the potential reasons for the observed heterogeneity in levels of concordance between YASA scoring and traditional manual scoring.

### YASA performance across sleep stages

The algorithm showed moderate-to-high agreement across most sleep stages, with the highest concordance observed for N3. This superior performance in N3 identification likely stems from the distinct EEG signatures of slow-wave sleep, characterized by high-amplitude, low-frequency (0.4–4 Hz) delta waves that predominantly occur during the first half of the night.

The lowest agreement rate occurred for N1, which converges with well-documented challenges even amongst expert raters in N1 identification for this transitional stage [8]. Identifying N1 is difficult in part because the EEG exhibits dynamic patterns that evolve throughout its duration (AASM) [5, 6]. Early N1 often retains residual 8–12 Hz alpha activity from wakefulness, whereas later N1 is dominated by 4–8 Hz theta activity. This creates inherent classification difficulties at both boundaries—with wakefulness (due to alpha persistence) and with N2 (due to shared theta activity). Though N2 epochs can be distinct when sleep spindles and K-complexes are present, in their absence, N1 and N2 display similar theta activity. In the current study, YASA frequently misclassified N1 epochs as either N2 (26% of epochs) or REM sleep (19% of epochs), likely due to intermittent spindle or K-complex events in N2 creating discontinuous boundaries, and REM’s mixed-frequency profile occasionally resembling transitional N1 patterns.

### YASA performance under sleep restriction conditions

During sleep-restricted nights, YASA exhibited lower overall agreement rates with manual scoring compared to normal sleep nights. One plausible explanation involves reduced classification tolerance due to compressed sleep duration [26]. Under normal sleep conditions, longer total sleep time and a larger number of total epochs may provide more data points across stages, increasing robustness against classification errors, making the algorithm's performance less sensitive to occasional misclassifications. In contrast, sleep restriction reduces total sleep duration without introducing the fragmentation patterns seen in pathological sleep. These conditions appear to challenge YASA’s performance, leading to slightly deflated overall performance, a moderate drop in REM performance, and a large drop in N1 performance. The misclassification analysis supported this reasoning. Sleep restriction resulted in more misclassifications of manually scored N1 and REM as being stage N2, that is, a stage that shows overlap in theta activity characteristics. Therefore, when the total sleep period is shortened, the algorithm has less opportunity to correct for or average out its inherent difficulties in distinguishing between stages with similar EEG features, leading to a lower overall agreement.

### YASA performance for sleep efficiency metrics

PSG-derived measures of sleep efficiency are important markers of overall sleep quality [27]. YASA and manual scoring methods often produced outcomes that were highly correlated; however, the different scoring methods consistently, albeit modestly, produced divergent absolute values for sleep onset latency and nighttime awakenings.

Regarding sleep onset latency, YASA indicated that participants fell asleep quicker than manual scoring estimates. Though YASA’s algorithm may be mistakenly identifying sleep onset too early, it is also possible that manual raters are hesitant to mark the first sleep epoch [8].

Regarding wake-related measures, manual staging yielded lower WASO durations and fewer arousal events compared to YASA. This discrepancy likely reflects fundamental differences in handling artifact and the interpretation of ambiguous electrophysiological events rather than human error. Human scorers often use contextual information to ignore EEG activity that appears to be movement or other artifacts, whereas YASA’s algorithm only uses the formal criteria for wakefulness or arousal (e.g., EEG amplitude and frequency). Though YASA’s approach provides more objective classifications, without having the context-specific information that is available to human raters, YASA seems likely to overestimate sleep fragmentation.

### Limitations and future directions

This study has limitations. First, the sample was composed of healthy young adults, which may lead to an overestimation of the accuracy of YASA classifications when applied to the broader population or clinical groups. Second, this study was conducted at a single center and utilized a single manual scorer as the reference. Third, the current work focused on sleep macro-architecture, and additional work will be needed to determine the validity of YASA’s automatic extraction of micro-architectural EEG features (e.g., spindle and slow-wave). Fourth, we investigated YASA’s performance under three common conditions for the field (adaptation, normal sleep, and mild sleep restriction), but additional work is needed to determine its efficacy under conditions of severe sleep deprivation, prolonged time-in-bed extension, and monophasic or polyphasic daytime napping, amongst other relevant conditions.

Nonetheless, the current findings can inform the future development of automated staging algorithms. The results demonstrate that YASA’s performance is challenged by the altered distribution and transitional dynamics of sleep stages that occur under sleep restriction. This condition, characterized by compressed sleep duration, may create EEG patterns that are underrepresented in existing training datasets. Therefore, a future direction would be to incorporate sleep-restricted data into YASA’s training and validation sets. Such an approach could enhance its performance, potentially through the development of adaptive thresholding for stage transitions under compressed sleep or improved feature extraction for discriminating between N1/N2 and N1/REM during sleep restriction. This modification could improve agreement rates while maintaining the algorithm’s efficiency advantages for large-scale studies.

## Conclusion

This study evaluated the YASA algorithm’s sleep stage performance in a multi-night study that included adaptation nights, normal sleep nights, and sleep-restricted nights. YASA exhibited good overall agreement with expert manual scoring. Caution is warranted, though, when interpreting YASA’s scoring of night awakenings and its classification of N1 and REM sleep, particularly during nights with short sleep duration (approximately 5 h). YASA is an efficient tool, but its optimal use in research or clinical settings will depend on the specific sleep parameters under investigation.

## Data Availability

The data are available at Open Science Framework (https://osf.io/qyp34/).
